# Small ubiquitin-related modifier (SUMO)ylation of SIRT1 mediates (-)-epicatechin inhibited- differentiation of cardiac fibroblasts into myofibroblasts

**DOI:** 10.1080/13880209.2022.2101672

**Published:** 2022-09-09

**Authors:** Yingchun Luo, Jing Lu, Zeng Wang, Lu Wang, Guodong Wu, Yuanyuan Guo, Zengxiang Dong

**Affiliations:** a Department of Cardiology, The First Affiliated Hospital, Harbin Medical University, Harbin, China; b Department of Pharmacy, The First Affiliated Hospital, Harbin Medical University, Harbin, China; c The Key Laboratory of Cardiovascular Disease Acousto-Optic Electromagnetic Diagnosis and Treatment in Heilongjiang Province, the First Affiliated Hospital, Harbin Medical University, Harbin, China

**Keywords:** Flavanol, cardiac fibrosis, sirtuin, SUMO1

## Abstract

**Context:**

(-)-Epicatechin (EPI) is a crucial substance involved in the protective effects of flavanol-rich foods. Previous studies have indicated EPI has a cardioprotective effect, but the molecular mechanisms in inhibition of cardiac fibrosis are unclear.

**Objective:**

We evaluated the effect of EPI in preventing cardiac fibrosis and the underlying molecular mechanism related to the SIRT1-SUMO1/AKT/GSK3β pathway.

**Materials and methods:**

Cardiac fibrosis mice model was established with transaortic constriction (TAC). Male C57BL/6 mice were randomly separated into 4 groups. Mice received 1 mg/kg/day of EPI or vehicle orally for 4 weeks. The acutely isolated cardiac fibroblasts were induced to myofibroblasts with 1 µM angiotensin II (Ang II). The cardiac function was measured with the ultrasonic instrument. Histological analysis of mice’s hearts was determined with H&E or Masson method. The protein level of fibrosis markers, SUMOylation of SIRT1, and AKT/GSK3β pathway were quantified by immunofluorescence and western blot.

**Results:**

EPI treatment (1 mg/kg/day) could reverse the TAC-induced decline in LVEF (TAC, 61.28% ± 1.33% vs. TAC + EPI, 74.00% ± 1.64%), LVFS (TAC, 28.16% ± 0.89% vs. TAC + EPI, 37.18% ± 1.29%). Meantime, we found that 10 µM EPI blocks Ang II-induced transformation of cardiac fibroblasts into myofibroblasts. The underlying mechanism of EPI-inhibited myofibroblasts transformation involves activation of SUMOylation of SIRT1 through SP1. Furthermore, SUMOylation of SIRT1 inhibited Ang II-induced fibrogenic effect via the AKT/GSK3β pathway.

**Conclusion:**

EPI plays a protective effect on cardiac fibrosis by regulating the SUMO1-dependent modulation of SIRT1, which provides a theoretical basis for use in clinical therapies.

## Introduction

Cardiac fibrosis, which can broadly be defined as the pathological transformation of fibroblasts into myofibroblasts, is a common pathophysiological process in cardiac remodelling and heart failure (Shah and Mann 2011; Braunwald 2013; Travers et al. 2016). Recently, there has been a potential treatment for inhibiting cardiac fibrosis by suppressing myofibroblasts transformation. (-)-Epicatechin (EPI) is an important chemical compound that exerts protective effects on the cardiovascular system, which is extracted from many flavanol-rich substances, including cocoa and green tea (Quine and Raghu 2005; Yamazaki et al. 2008, 2010; Taub et al. 2012). Continuous EPI treatment had a blood pressure-lowering effect in young male borderline hypertensive rats (Kluknavsky et al. 2020). Treatment with EPI has capable of reducing myocardial fibrosis in high glucose-stimulated cardiac fibroblasts (Garate-Carrillo et al. 2021). The above studies focussed primarily on the role of EPI in cardiac function; however, the specific action of EPI on cardiac fibrosis remains unclear, and the exact mechanism of the EPI regulating pathway in cardiac fibroblasts needs to be determined.

SIRT1, a member of the sirtuin family of nicotinamide adenine dinucleotide (NAD^+^)- dependent histone deacetylases, is not only involved in regulating oxidative stress and inflammatory but also played a protective role in organ fibrosis (Haigis and Sinclair 2010; Zeng et al. 2017; Shaikh et al. 2019). The activation of SIRT1 could prevent cardiac fibrosis induced by isoproterenol via the regulation of epithelial-to-mesenchymal transition (Liu et al. 2019). The expression of SIRT1 is controlled at both the transcriptional and post-transcriptional regulation levels; however, the post-translational mechanisms that regulate SIRT1 stability remain unclear.

Small ubiquitin-related modifier (SUMO) participated in post-transcriptional regulation, nucleo-cytoplasmic transport, and DNA repair (Breucker and Pichler 2019). SUMOylation of SERCA2a inhibited cardiac dysfunction induced by pressure overload (Kho et al. 2011). Of note, SUMOylation of SIRT1 regulated cellular senescence and stabilized protein levels (Han et al. 2016). Moreover, by activating the AKT pathway, inhibition of SENP2-mediated AKT deSUMOylation promotes cardiac regeneration (Chen et al. 2021). However, whether SUMOylation of SIRT1 participates in EPI-inhibited cardiac fibrosis is unclear.

Therefore, we aimed to evaluate the protective effect of EPI on cardiac fibrosis and elucidate the exact mechanism. Through establishing the cardiac fibrosis model *in vivo* and *in vitro*, we demonstrated an anti-fibrotic effect of EPI on cardiac fibrosis and the role of SUMO1-dependent modulation of SIRT1 participated in EPI inhibiting cardiac fibrosis.

## Materials and methods

### Animal model and treatment

Male C57BL/6 mice weighing between 22–28 g and aged 8 weeks were fed through a freely available diet for 7 days of acclimatization under normal room temperature and humidity conditions. For all *in vivo* studies, male C57BL/6 mice were used with at least five mice per genotype. Mice were then randomly separated into sham/control, EPI (1 mg/kg/day), TAC, and TAC + EPI (1 mg/kg/day) groups. The dose selection of EPI was confirmed by a previous study (Li et al. 2018). Tribromoethyl alcohol (20 mg/kg, i.p., MCE, USA) was used to anaesthetize mice. TAC mouse model was conducted as a previous study (Qin et al. 2015). Intragastric administration of EPI was conducted in mice for 28 days. Animal experiments were approved by the Institutional Animal Care and Use Committee of First Affiliated Hospital, Harbin Medical University (approval No. 2020099), and complied with the Guide for the Use and Care of Laboratory Animals published by the National Institutes of Health (NIH Publication No. 85-23, revised 1996).

### Echocardiographic measurement

Echocardiography of cardiac structure and function was determined as previously reported (Luo et al. 2015). Echocardiographic measurements were conducted with ultrasonic echocardiographic instruments (Visualsonic Vevo 1100, Canada). The cardiac parameters of five mice were measured in each group after 28 days of TAC or treatment.

### Histological analysis

Histological analysis of mouse hearts was determined with H&E or Masson method. The heart tissues were fixed in 4% paraformaldehyde for 2 days and then embedded in paraffin and sliced into 4 μm sections. H&E staining kit (Solarbio, Beijing, China) and Masson’s trichrome stain kit (Solarbio, Beijing, China) were used to measure cardiomyocyte area and cardiac fibrosis. The myocardial sections were observed by microscope (Carl Zeiss Co. Ltd., USA). Collagen deposition was evaluated with collagen volume fraction (CVF). The heart weight/body weight ratio (HW/BW) index was calculated using the mouse heart weight to body weight ratio.

### Immunohistochemistry assays (IHC)

Mouse heart tissue sections were deparaffinized in xylene series and rehydrated through ethanol series. Sections were subjected to antigen retrieval in citrate buffer before the addition of SIRT1 primary antibody (Proteintech, Wuhan, China) overnight at 4 °C. The following day, the sections were incubated with horseradish peroxidase (HRP)-conjugated anti-mouse antibody and stained with diaminobenzidine (DAB). Thereafter, incubated with the 3,3′-diaminobenzidine solution for 10 sec. Images were taken with the microscope (Carl Zeiss Co. Ltd., USA).

### Cell culture

Cardiac fibroblasts (CFs) and cardiomyocytes (CMs) were acutely isolated from neonatal SD rats (1-3-day-old). CFs, CMs, and H9C2 cells were cultured under routine conditions (37 °C, 5% CO_2_). The cells were divided into different groups and subjected to experimental procedures at 80% confluence. According to the experimental group, CFs, CMs, and H9C2 cells were treated with 1 µM Ang II, 10 µM EPI, and 0.05 µM MTM (SP1 inhibitor) for 24 h.

### Small interference RNA (Si-RNA) transfection

CFs were transfected with SIRT1 Si-RNA or NC Si-RNA (Genechem, Shanghai, China). The SIRT1 and NC Si-RNA sequences: SIRT1, sense 5′-GATCCCACCCTGTAAAGCTTTCAGAAACTCGAGTTTCTGAAAGCTTTACAGGGTTTTTTGGAT-3′ and antisense 5′-AGCTATCCAAAAAACCCTGTAAAGCTTTCAGAAACTCGAGTTTCTGAAAGCTTTACAGGGTGG-3′; and NC, sense 5′-GATCCCTTCTCCGAACGTGTCACGTCTCGAGACGTGACACGTTCGGAGAATTTTTGGAT-3′- and antisense 5′-AGCTATCCAAAAATTCTCCGAACGTGTCACGTCTCGAGACGTGACACGTTCGGAGAAGG-3′. After CFs had grown to 80% confluence on a 60 mm dish, transfection was accomplished by using GoldenTran®-DR.

### Western blot analysis

Cell protein was extracted from CFs, CMs, and H9C2 cells under different conditions for analysis. The blots were probed with primary antibodies including α-SMA (Santa Cruz, USA), COLI /COLIII (Santa Cruz, TX, USA), SP1 (Santa Cruz, TX, USA), SIRT1 (Abcam, MA, USA), SUMO1 (Santa Cruz, TX, USA), Phospho-AKT (Santa Cruz, TX, USA), Phospho-GSK3β (Santa Cruz, TX, USA), β-actin (ZSGB-Biotech, Beijing, China) and GAPDH (ZSGB-Biotech, Beijing, China). The blots were probed with the secondary antibody (ZSGB-Biotech, Beijing, China). The bands were detected by an imaging instrument (LI-COR Biosciences, NE, USA).

### Co-immunoprecipitation (Co-IP)

CFs were lysed with the buffer containing a protease inhibitor after washing. The supernatants of cell lysates were obtained with centrifuging at 15,000 rpm at 4 °C for 15 min, incubated with SIRT1 polyclonal antibody at 4 °C for 3 h. Then, 30 µL magnetic beads (Biomake, TX, USA) were added to the samples at 4 °C for 12 h. Co-IP proteins were eluted from the magnetic beads and immunoblotted with antibodies against SIRT1 or SUMO1.

### Immunofluorescence assay

Briefly, CFs were stained with α-SMA antibody for 12 h. Cells were washed with PBS. Then, the secondary antibody (Molecular Probes, Invitrogen) was added for 1 h. DAPI was added for 3 min. Immunofluorescence was detected by microscope (Carl Zeiss, USA). The magnification is ×200.

### Statistical analysis

Results were shown as the mean ± SEM and analysed with SPSS 20.0 statistical software. Difference comparisons were determined using one-way ANOVA and considered statistically significant when *p*< 0.05.

## Results

### EPI prevents cardiac fibrosis

**Figure 1.**
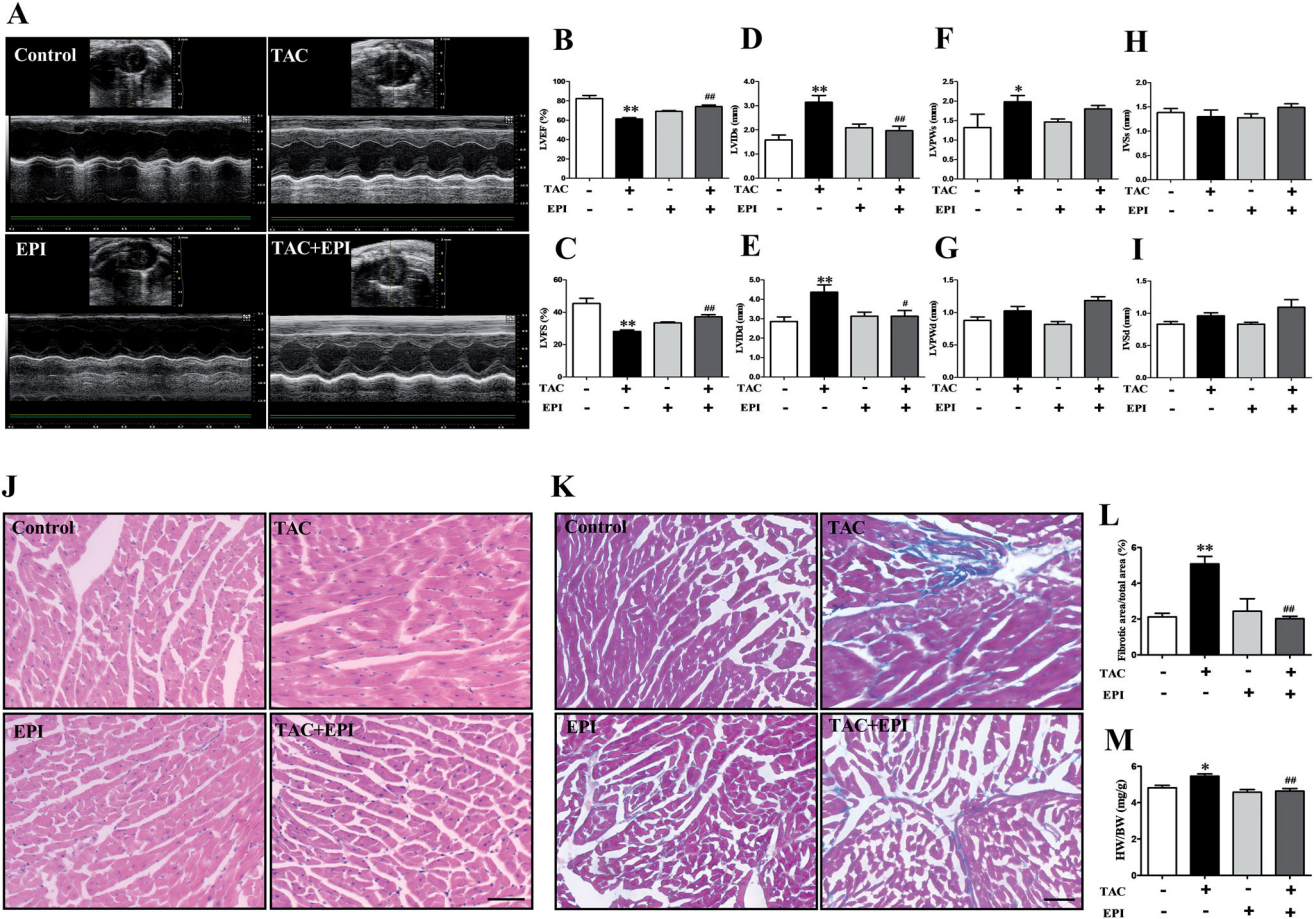
The effect of EPI on cardiac function and myocardial structure in mice. (A) Representative cardiac echocardiography of the indicated groups. (B-I) LVEF: left ventricular ejection fraction, LVFS: left ventricular fractional shortening, LVIDs: left ventricular systolic internal diameter, LVIDd: left ventricular diastolic internal diameter, LVPWs: Left ventricular posterior wall thickness in systole, LVPWd: left ventricular posterior wall thickness in diastole, IVSs: interventricular septal thickness at end systole, IVSd: interventricular septal thickness at end-diastole. Statistical analysis of LVEF, LVFS, LVIDs, LVIDd, LVPWs, LVPWd, IVSs, IVSd (*n* = 5). Representative graph of H&E (J) and Masson (K). The magnification is ×200, scale bar = 100 μm. Statistical analysis of collagen deposition (L) and HW/BW index (M). Data are shown as mean ± SEM (*n* = 6). *p < 0.05, **p < 0.01 compared with control group; ^#^*p* < 0.05, ^##^*p* < 0.01 compared with TAC group.

To examine whether EPI protects the heart against cardiac fibrosis, we first established the TAC mouse models. Then intragastric administration of EPI was conducted in mice for 28 days after established TAC models. Echocardiography was used to monitor cardiac function (Figure 1(A)). LVEF, LVFS, LVIDs, LVIDd, LVPWs, LVPWd, IVSs, and IVSd were detected (Figure 1(B–I)). Compared to the control group, TAC decreased LVEF, LVFS, and increased LVIDs, LVIDd, which were ameliorated by EPI treatment.

The protective effect of EPI on the TAC-induced cardiac structure of mice was determined. The results showed that collagen content was increased in the heart tissues of the TAC group, which was significantly reduced by EPI treatment (Figure 1(J–L)). In addition, the administration of EPI substantially reduced HW/BW index in the TAC group (Figure 1(M)).

### EPI blocks Ang II/TGF-β1/SMAD3-induced myofibroblasts transformation

**Figure 2.**
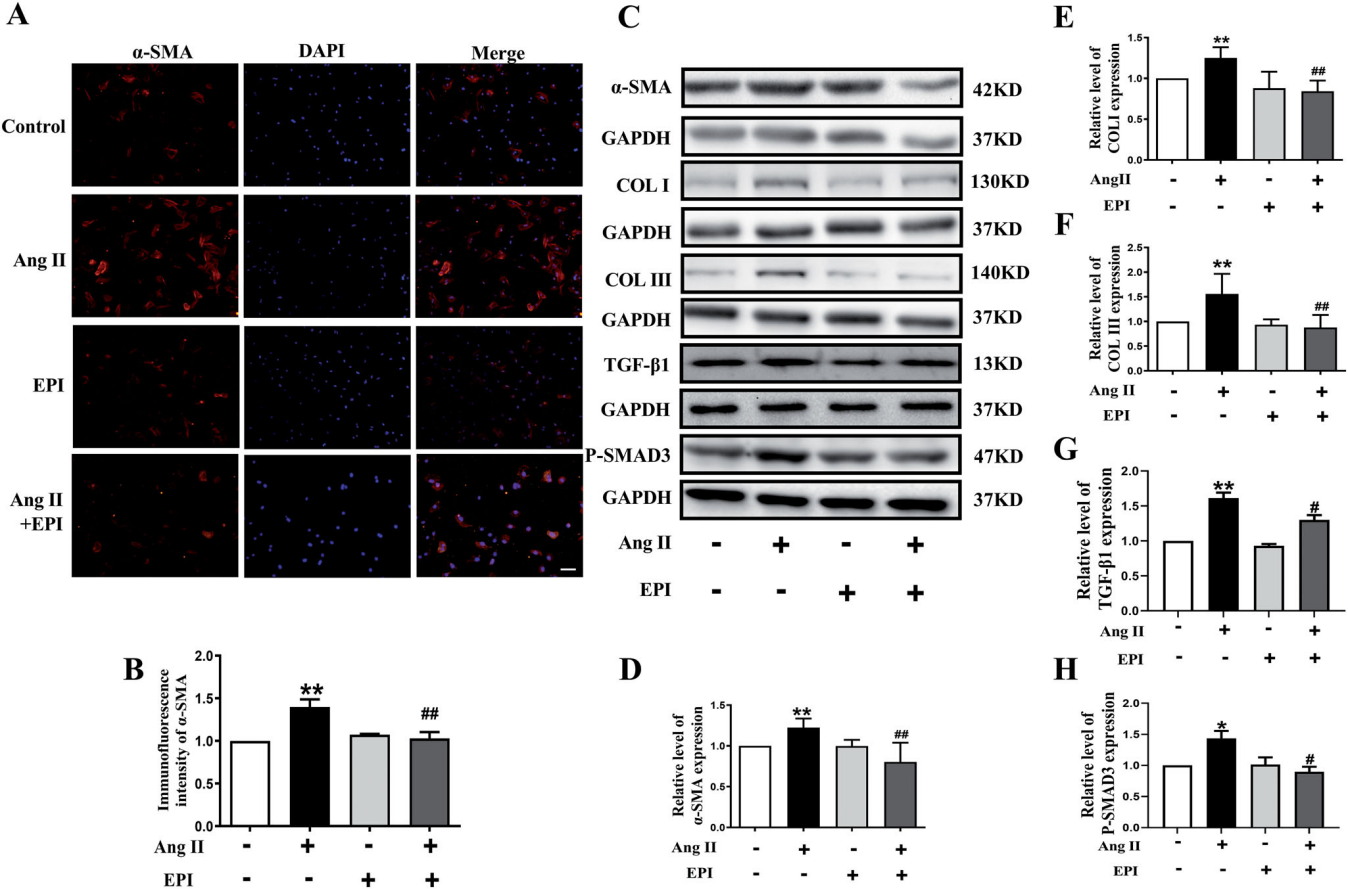
EPI blocks Ang II-induced myofibroblast transformation. (A) CFs stained with α-SMA (red) and DAPI (blue) antibody in each group. (B) Statistical analysis of α-SMA. The magnification is ×200, scale bar = 50 μm. (C) Representative western blot bands of α-SMA, COL I, COL III, TGF-β1 and P-SMAD3 after incubation with EPI or-/and Ang II in CFs. (D-H) Statistical analysis of α-SMA, COL I/COL III, TGF-β1 and P-SMAD3. Data are shown as mean ± SEM (*n* = 3–6). *p < 0.05, **p < 0.01 compared with the control group; ^#^*p* < 0.05, ^##^*p* < 0.01 compared with the Ang II group.

To examine whether EPI protects CFs against Ang II-induced myofibroblasts transformation, we used primary cultured neonatal rat cardiac fibroblast for *in vitro* models of cardiac fibrosis. Cells were incubated using Ang II or/and EPI for 24 h. As shown in Figure 2(A,B), the α-SMA-positive area was significantly increased in the Ang II-treated group, and EPI inhibited Ang II-induced fibroblast transformation.

To further evaluate the protective effect of EPI on cardiac fibrosis, protein of α-SMA, Collagen I/III (COL I/III), TGF-β1 and P-SMAD3 were detected by western blot (Figure 2(C–H)). As we expected, Ang II increased the protein expression of α-SMA and COL I/III, TGF-β1 and P-SMAD3. EPI efficiently inhibited these changes, suggesting that EPI could mitigate the myofibroblasts transformation and collagen synthesis induced by Ang II.

### EPI activates SUMOylation of SIRT1

The SUMOylation of SIRT1 played a vital role in regulating cellular physiological functions (Han et al. 2016). To examine EPI whether protected the heart through SUMOylation of SIRT1, we firstly detected the SIRT1 and SUMO1 expression in mice heart tissues by IHC assay. Meanwhile, cells were incubated through Ang II or/and EPI for 24 h.

**Figure 3.**
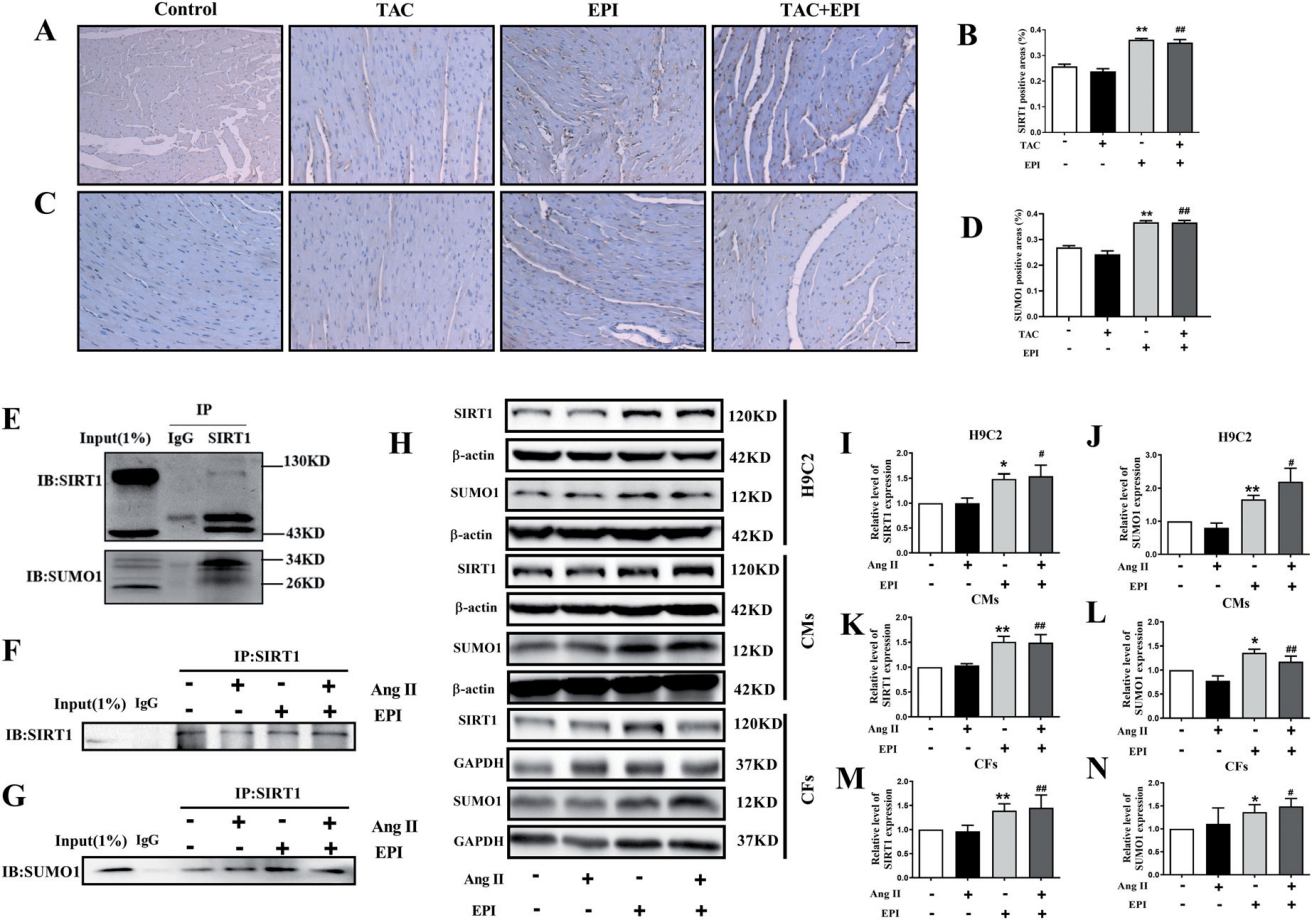
EPI activates SIRT1-SUMO1. Representative images of IHC staining in mice heart with SIRT1 (A) and SUMO1 (C) antibody. The magnification is ×200, scale bar = 50 μm. Statistical analysis of SIRT1 (B) and SUMO1 (D) (*n* = 5). (E) Representative immunoblots of SIRT1-SUMOylation in CFs (*n* = 3). (F-G) Representative immunoblots of SIRT1-SUMOylation after incubation with EPI or /and Ang II in CFs (*n* = 3). (H) Representative western blot bands of SIRT1-SUMO1 after incubation with EPI or /and Ang II in H9C2, CMs and CFs. (I-N) Statistics of SIRT1-SUMO1 expression in H9C2, CMs and CFs. Data are shown as mean ± SEM (*n* = 6). *p < 0.05, **p < 0.01 compared with the control group; ^#^*p* < 0.05, ^##^*p* < 0.01 compared with the Ang II/TAC group.

The IHC assay shows that SIRT1 and SUMO1 protein are expressed at lower levels in EPI and TAC + EPI groups, whereas Control and TAC groups are weakly detected of SIRT1 and SUMO1 in heart tissues (Figure 3(A–D)). We then examined whether SIRT1 is indeed SUMOylated in CFs. As shown in Figure 3(E–G), the Western Blot bands represented SUMOylated SIRT1, and EPI increased the SUMOylation of SIRT1. In addition, for further confirmation of the effect of EPI on SIRT1-SUMO1, the protein expression of SIRT1-SUMO1 was detected in H9C2, CMs, and CFs that incubated with EPI (Figure 3(H)). As shown in Figure 3(I–N), the results indicated that the protein expression of SIRT1-SUMO1 was increased in the EPI treatment group. The data indicated that EPI could increase SIRT1-SUMO1 protein expression in both H9C2, CMs and CFs.

### SIRT1-SUMO1 participates in EPI-induced activation of AKT/GSK3β

**Figure 4.**
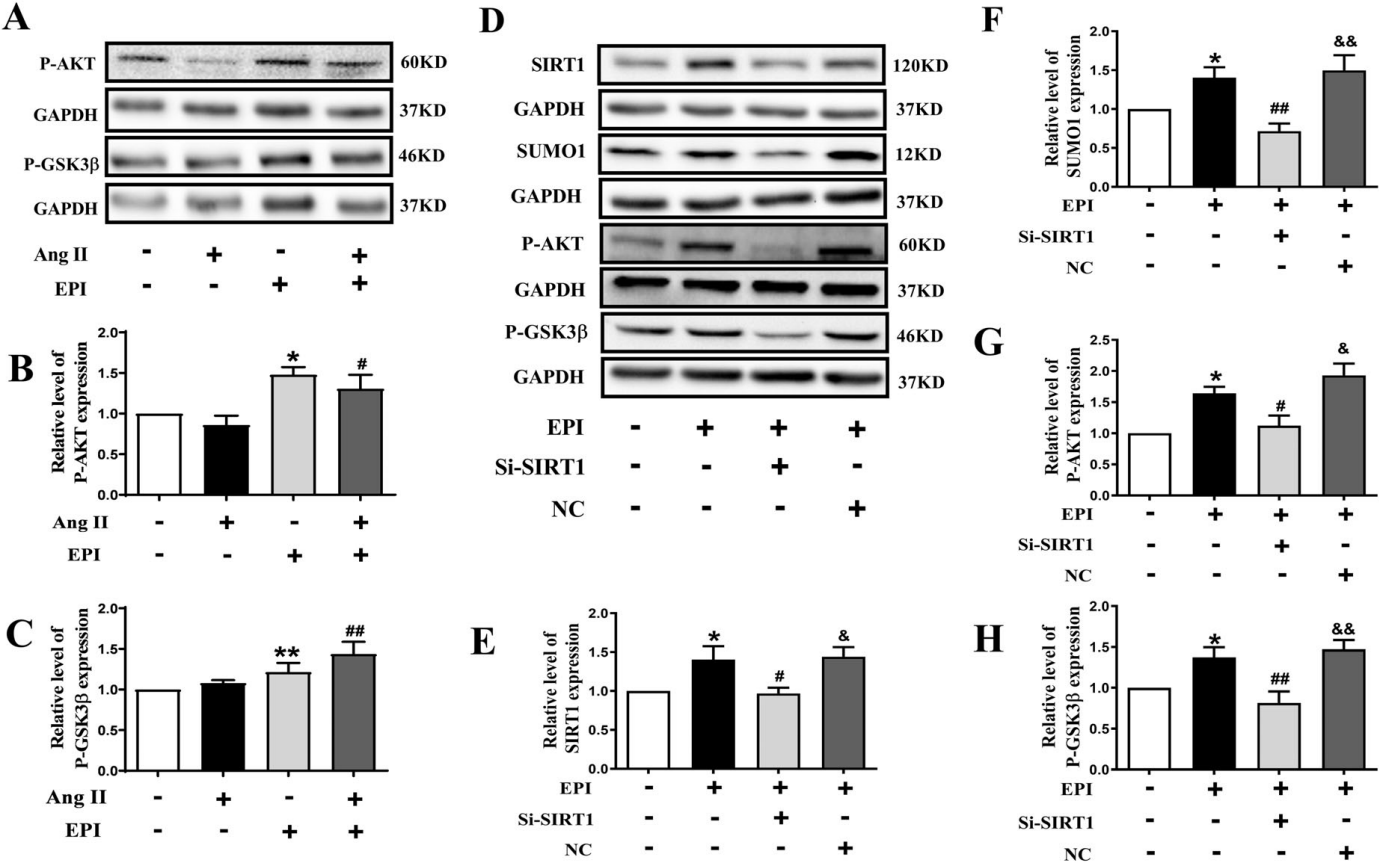
The effect of SIRT1-SUMO1 on EPI-induced protein levels of P-AKT and P-GSK3β. (A) Representative western blot bands of P-AKT and P-GSK3β. (B-C) Statistics of P-AKT/P-GSK3β expression in CFs. (D) Representative western blot bands of SIRT1-SUMO1, P-AKT, and P-GSK3β after incubation with EPI, Si-SIRT1, and NC in CFs. (E-H) Statistics of SIRT1, SUMO1, P-AKT, and P-GSK3β protein expression levels. Data are shown as mean ± SEM (*n* = 6). *p < 0.05, **p < 0.01 compared with control group; ^#^*p* < 0.05, ^##^*p* < 0.01 compared with EPI group; ^&^*p* < 0.05, ^&&^*p* < 0.01 compared with EPI + Si-SIRT1 group.

We explored whether EPI affected the activation of AKT and GSK3β. As indicated in Figure 4(A–C), EPI increased the protein of P-AKT and P-GSK3β in CFs. These data suggested that EPI might activate the SIRT1-SUMO1/AKT/GSK3β pathway to inhibit cardiac fibrosis.

We explored whether SIRT1-SUMO1 indeed participated in regulating of AKT/GSK3β pathway. As shown in Figure 4(D–H), we used the small interfering RNAs approach to knockdown SIRT1, which blocked SIRT1 and SUMO1 proteins increased by EPI. As we know, SIRT1 could regulate the activation of GSK3β (Yang et al. 2020). We tested whether knockdown of SIRT1 could affect activation of AKT/GSK3β induced by EPI. As we expected, the knockdown of SIRT1 significantly decreased the protein level of P-AKT and P-GSK3β. These data illustrated that EPI-induced activation of AKT and GSK3β was mediated by SIRT1-SUMO1.

### SIRT1-SUMO1 participates in EPI-inhibited myofibroblasts transformation

**Figure 5.**
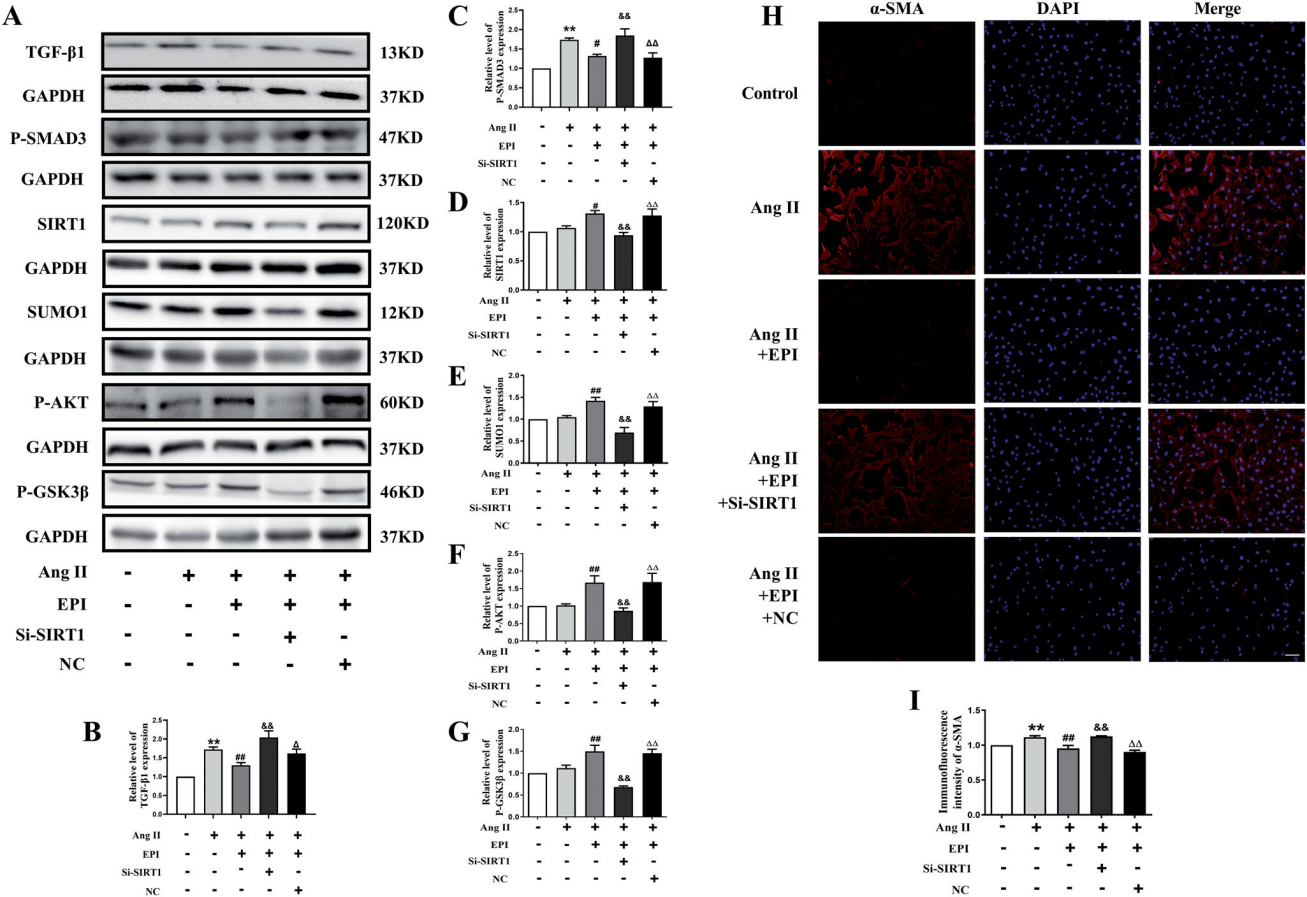
The effect of SIRT1-SUMO1 on myofibroblast transformation and protein levels of P-AKT/P-GSK3β. (A) Western blot bands of TGF-β1, P-SMAD3, SIRT1, SUMO1, P-AKT and P-GSK3β after incubation with Ang II, EPI, Si-SIRT1, and Si-N in CFs. (B-G) Statistics of TGF-β1, P-SMAD3, SIRT1, SUMO1, P-AKT, and P-GSK3β expression in each group. (H) CFs stained with α-SMA (red) and DAPI (blue) antibody in each group. (I) Statistical analysis of α-SMA. The magnification is ×200, scale bar = 50 μm. Data are shown as mean ± SEM (*n* = 3–6). ** *p*< 0.01 compared with control group; ^#^*p* < 0.05, ^##^*p* < 0.01 compared with Ang II group; ^&&^ *p*< 0.01 compared with Ang II + EPI group; ^△^* p*< 0.05, ^△△^*p* < 0.01 compared with Ang II + EPI + Si-SIRT1 group.

Furthermore, as SIRT1-SUMO1 has been shown to participate in EPI-induced activation of AKT and GSK3β, we tested whether SIRT1-SUMO1 was involved in EPI-inhibited myofibroblasts transformation. EPI decreased TGF-β1 and P-SMAD3 levels which increased by Si-SIRT1 (Figure 5(A–C)), increased SIRT1 and SUMO1 levels which decreased by Si-SIRT1 (Figure 5(D,E)), significantly decreased protein levels of P-AKT and P-GSK3β (Figure 5(F,G)), and blocked EPI-inhibited myofibroblasts transformation (Figure 5(H,I)). These results suggested that SIRT1-SUMO1 was essential for EPI-induced activation of AKT and GSK3β, which was also required for EPI-inhibited myofibroblasts transformation.

**Figure 6.**
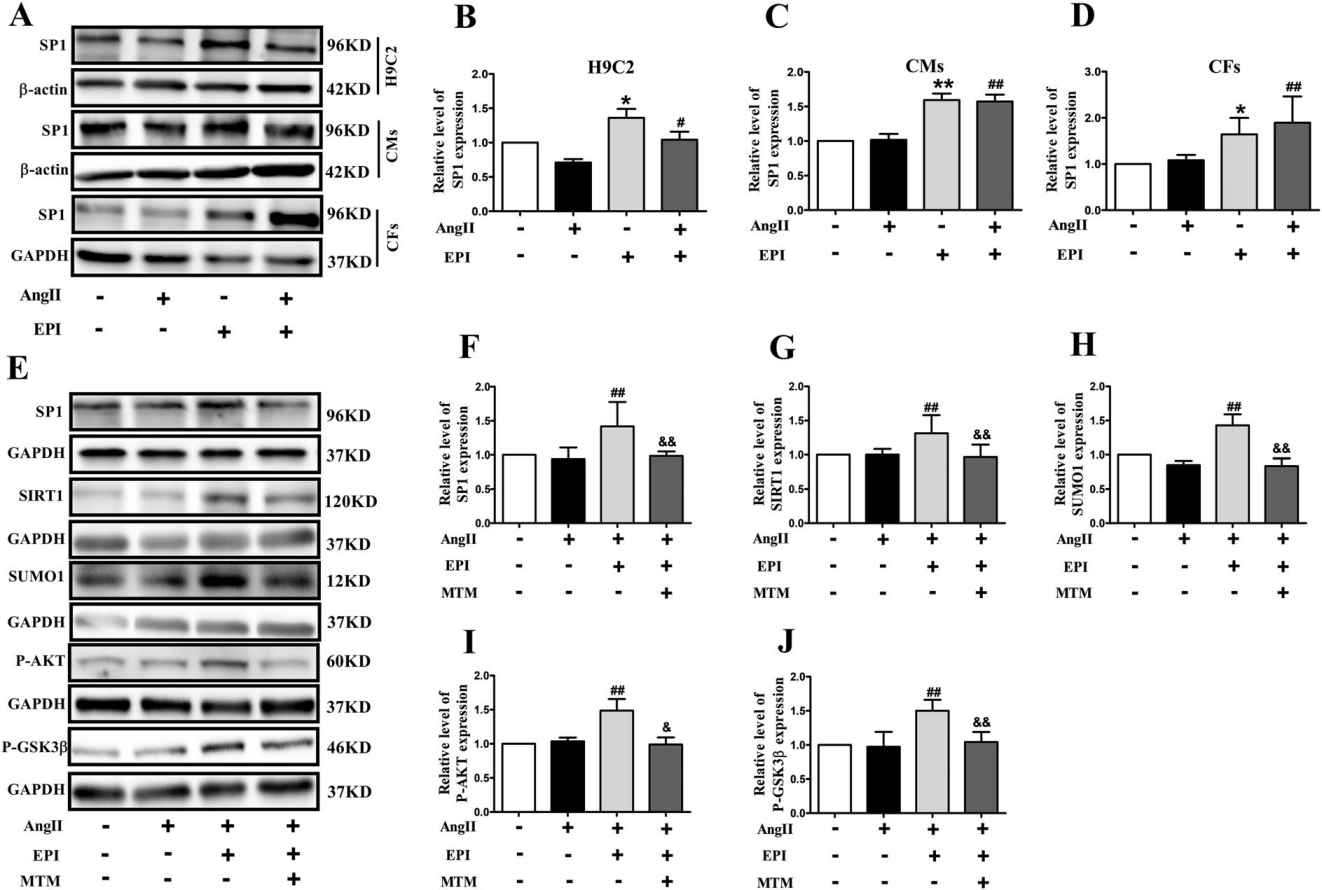
The effect of SP1 on protein levels of SIRT1-SUMO1 /AKT/GSK3β pathway. (A) Representative western blot bands of SP1 in H9C2, CMs, and CFs. (B-D) Statistics of SP1 expression in H9C2, CMs, and CFs. (E) Representative western blot bands of SP1, SIRT1, SUMO1, P-AKT, and P-GSK3β after incubation with Ang II, EPI, and MTM in CFs. (F-J) Statistics of SP1, SIRT1-SUMO1, P-AKT, P-GSK3β expression in CFs. Data are shown as mean ± SEM (*n* = 6). *p < 0.05, **p < 0.01 compared with control group; ^#^*p* < 0.05, ^##^*p* < 0.01 compared with Ang II group; ^&^*p* < 0.05, ^&&^*p* < 0.01 compared with Ang II + EPI group.

**Figure 7.**
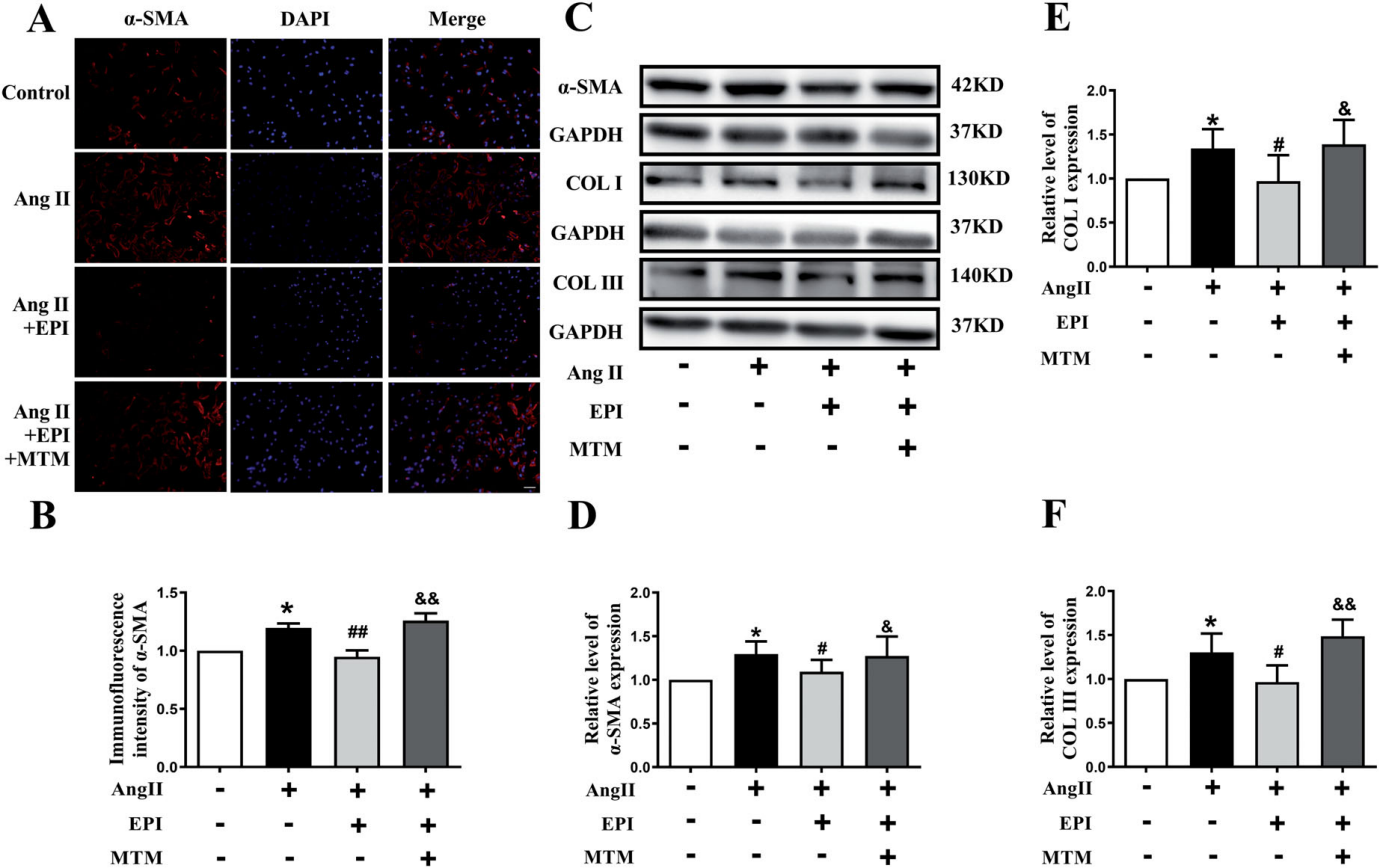
The effect of SP1 on Ang II-induced myofibroblast transformation. (A) CFs stained with α-SMA (red) and DAPI (blue) antibody in each group. (B) Statistical analysis of α-SMA. The magnification is ×200, scale bar = 50 μm. (C) Western blot bands of α-SMA, COLI/COLIII. (D-F) Statistical analysis of α-SMA, COL I/COL III. Data are shown as mean ± SEM (*n* = 6). *p < 0.05 compared with control group; ^#^*p* < 0.05, ^##^*p* < 0.01 compared with Ang II group; ^&^*p* < 0.05, ^&&^*p* < 0.01 compared with Ang II + EPI group.

Transcription factor SP1 is considered as a constitutive activator of housekeeping genes. EPI was known to regulate activity of transcription factor SP1 (Feng et al. 2019). As indicated in Figure 6(A–D), EPI could increase protein level of SP1 in both H9C2, CMs and CFs. EPI increased expression of SP1, SIRT1, SUMO1, P-AKT and P-GSK3β were consistent with above observation, MTM (SP1 inhibitor) completely blocked effect of EPI (Figure 6(E–J)). Furthermore, CFs were preincubated with MTM which showed an obvious increase of the expression levels of α-SMA and COLI/III in CFs (Figure 7(A–F)). These data suggested that SP1 was required for EPI-induced SIRT1-SUMO1 activation.

## Discussion

A diet rich in flavanols has a protective effect on cardiovascular health and function as an antioxidant and anti-inflammatory (Yamazaki et al. 2010; Wu et al. 2017; Kang et al. 2020; Leyva-Soto et al. 2021). EPI is extracted from the flavanol-rich plant and has been validated as a dietary supplement in mitigating vascular inflammation and oxidative stress. EPI treatment has been confirmed that can protect the heart against myocardial ischaemia induced-cardiac injury and protect the cardiovascular through reducing arginase expression and increasing NOS expression (Li et al. 2018; Ortiz-Vilchis et al. 2018; MacRae et al. 2019). Furthermore, EGCG treatment could ameliorate cardiac hypertrophy and fibrosis (Cai et al. 2013; Muhammed et al. 2018). In our present study, EPI was verified to improve heart function and reduce HW/BW ratio to protect the heart against cardiac fibrosis by the TAC mouse model. Furthermore, H&E and Masson staining results show the cardiovascular protective effect of EPI. The immunohistochemical analysis results show that the SIRT1 protein expression in the hearts of the TAC mice is upregulated by EPI treatment. EPI treatment decreased the protein level of cardiac fibrosis biomarkers such as COLI, COLIII, and α-SMA. Therefore, EPI blocked TAC or Ang II-induced myofibroblasts transformation and collagen synthesis.

SIRT1 could inhibit oxidative stress, inflammatory and attenuate cardiac fibrosis (Liu et al. 2019). SIRT1 was indeed activated by SUMOylation to stabilize its protein levels (Han et al. 2016). The SUMOylation of SIRT1 is involved in inhibition of cellular senescence and acted as a cardioprotective factor against MI/R injury (Yang et al. 2007; Han et al. 2016; Chen et al. 2019). In our study, we found that SIRT1 and SUMO1 participated in EPI-inhibited myofibroblasts transformation and protein levels were upregulated by EPI. The Co-IP results show that SIRT1 could be SUMOylated, and SUMOylation of SIRT1 participated in EPI-inhibited myofibroblasts transformation. Therefore, we found that EPI-inhibited myofibroblasts transformation might involve activation of SUMOylation of SIRT1.

In addition, the transcription factor SP1 can function in the transcriptional regulation of many genes in positive or negative ways (Schäfer et al. 2003; Chou et al. 2005). Previous studies have revealed that oxidation of SP1 was an essential element for the transcriptional regulation of SUMO2 and SUMO3 (Sang et al. 2011). Our research showed that EPI increased protein expression of SP1, SIRT1-SUMO1 in CFs. We also observed that MTM blocked the activation of SIRT1-SUMO1 induced by EPI. Furthermore, MTM blocked the inhibition of EPI on myofibroblasts transformation and collagen synthesis *in vitro*. The results indicated that SP1 participated in EPI-induced SIRT1-SUMO activation.

Previous studies found that GSK3β mediated the regulation of several signalling proteins (MacAulay and Woodgett 2008; Zhou et al. 2016). GSK3β also participated in activating CFs and regulated cardiac remodelling function (Liu et al. 2016). AKT/GSK3β pathway played a crucial role in regulating cell proliferation and apoptosis (Dai et al. 2016). Furthermore, activating SIRT1 and AKT signalling could prevent cardiac fibrosis in post-myocardial infarction (Jia et al. 2019). The western blot results show that knockdown SIRT1 significantly decreased the protein level of P-AKT and P-GSK3β and blocked EPI-inhibited myofibroblasts transformation in CFs. These data indicated that SIRT1-SUMO1 was involved in EPI-induced activation of AKT/GSK3β, which was also required for EPI-inhibited myofibroblasts transformation in CFs. In our study, Ang II did not affect SIRT1-SUMO1/AKT/ GSK3β pathway. The previous study indicated that Ang II could induce myofibroblasts transformation through the TGFβ1-SMAD3 pathway (Zhou et al. 2017). Moreover, the activation of GSK3β could inhibit the TGFβ1-SMAD3 pathway (Lal et al. 2014). So, our results indicate that EPI-induced activation of AKT/GSK3β inhibits the Ang II/TGFβ1-SMAD3 pathway for reducing myofibroblasts transformation. Our data indicated that EPI not only increased expression of SUMO1, but also increased the expression of SIRT1. The follow-up mechanism needs to be determined.

## Conclusions

Our study indicates that EPI can prevent cardiac fibrosis. SUMO1-dependent modulation of SIRT1 mediates EPI-inhibited cardiac fibrosis. These findings will supply a new agent and mechanism of action for treating cardiac fibrosis in the future.

## Data Availability

The datasets used and/or analysed during the current study are available from the corresponding author on reasonable request.
